# Supplementary material for: Macrophage Cholesterol Efflux Downregulation Is Not Associated with Abdominal Aortic Aneurysm (AAA) Progression
Source: Biomolecules. 2020 Apr 24;10(4):662. doi: 10.3390/biom10040662 (PMC7226271; doi:10.3390/biom10040662)
Supplement: Supplementary file 1 [file biomolecules-10-00662-s001.pdf]

biomolecules

MDPI

Supplementary Material

Macrophage Cholesterol Efflux Downregulation is Not Associated with Abdominal Aortic Aneurysm (AAA) Progression

Marina Canyelles[1,2], Mireia Tondo [1], Jes S. Lindholt [3], David Santos [4,5], Irati Fernández-Alonso [4], David de Gonzalo-Calvo [4,6,7], Luis Miguel Blanco-Colio [7,8], Joan Carles Escolà-Gil [2,4,5,*,†], José Luís Martín-Ventura [7,8,*,†] and Francisco Blanco-Vaca [1,2,5,*,†]

1 Servei de Bioquímica, Hospital de la Santa Creu i Sant Pau, IIB Sant Pau, 08041 Barcelona, Spain; mcanyelles@santpau.cat (M.C.); mtondo@santpau.cat (M.T.); fblancova@santpau.cat (F.B.-V.)
2 Departament de Bioquímica i Biologia Molecular, Universitat Autònoma de Barcelona, 08041 Barcelona, Spain
3 Centre of Individualized Medicine in Arterial Disease (CIMA), Department of Cardiology, Odense University Hospital, 5000 Odense, Denmark; Jes.Sanddal.Lindholt@rsyd.dk
4 Institut de Recerca de l'Hospital de la Santa Creu i Sant Pau- IIB Sant Pau, 08041 Barcelona, Spain; dsantos@santpau.cat (D.S.); irati.fernandezalonso@gmail.com (I.F.-A.); david.degonzalo@gmail.com (D.d.G.-C.)
5 CIBER de Diabetes y Enfermedades Metabólicas Asociadas (CIBERDEM), 28029 Madrid, Spain
6 Institute of Biomedical Research of Barcelona (IIBB)–Spanish National Research Council (CSIC), 08036 Barcelona, Spain
7 CIBER de Cardiovascular (CIBERCV), Instituto de Salud Carlos III, 28029 Madrid, Spain; lblanco@fjd.es
8 IIS-Fundación Jiménez Díaz, 28040 Madrid, Spain
* Correspondence: jescola@santpau.cat (J.C.E.-G.); JLmartin@fjd.es (J.L.M.-V.); fblancova@santpau.cat (F.B.-V.)
† Senior authors.

Supplementary Materials Table S1. Clinical and biochemical parameters of small/medium size AAA patients.

	Low progression (n = 26)	Medium progression (n = 29)	High progression (n = 26)	ANOVA or chi-square p value
**Age (years)**	69.88 ± 3.14	69.46 ± 2.69	69.60 ± 2.68	ns
**BMI (%)**	28.45 ± 3.21	27.43 ± 2.90	26.31 ± 4.09	ns
**Total Cholesterol (mmol/L)**	4.78 ± 0.96	4.85 ± 0.81	5.01 ± 0.89	ns
**ApoA-I (g/L)**	1.54 ± 0.37	1.66 ± 0.35	1.55 ± 0.19	ns
**TG (mmol/L)**	1.45 ± 0.52	1.40 ± 0.76	1.46 ± 0.69	ns
**HDLc (mmol/L)**	1.04 ± 0.39	1.17 ± 0.44	1.08 ± 0.35	ns
**LDLc (mmol/L)**	3.08 ± 0.92	3.10 ± 0.89	3.23 ± 0.93	ns
**VLDLc (mmol/L)**	0.66 ± 0.24	0.58 ± 0.27	0.70 ± 0.30	ns
**Aortic diameter (mm)**	36.00 ± 4.17	35.67 ± 4.90	37.50 ± 4.41	ns
**Diastolic Blood Pressure (mm Hg)**	85.76 ± 12.25	89.70 ± 12.60	88.43 ± 12.16	ns
**Lowest ABI**	0.96 ± 0.19	0.94 ± 0.18	0.95 ± 0.19	ns
**Smoke**	9 (35 %)	10 (36 %)	13 (52 %)	ns
**Diabetes**	5 (19 %)	4 (14 %)	1 (4 %)	ns

**Arterial Hypertension**	15 (58 %)	17 (61 %)	9 (36 %)	ns
**Previous CVD**	4 (15 %)	5 (18 %)	2 (8 %)	ns
**Statins use**	16 (62 %)	18 (64 %)	7 (28 %)	< 0.05
**Low Dose Aspirin**	16 (62 %)	14 (50 %)	2 (20 %)	< 0.01

Results expressed as mean ± standard deviation (SD), ns = non-significant

Supplementary Materials Table S2. One-way ANCOVA of MCE in all subjects adjusted for age, BMI and DBP

Tests of Between-Subjects Effect<br>Dependent Variable: Efflux					
**Source**	**Type III Sum of Squares**	**df**	**Mean Square**	**F**	**p value**
Corrected Model	395.662[a]	5	79.132	7.143	0.000
Intercept	51.861	1	51.861	4.681	0.032
Age	5.697	1	5.697	0.514	0.475
BMI	190.872	1	190.872	17.230	0.000
DBP	0.027	1	0.027	0.002	0.961
Group	102.848	2	51.424	4.642	0.011
Error	1528.785	138	11.078		
Total	20793.361	144			
Corrected Total	1924.447	143			

a. R Squared = 0.206 (Adjusted R Squared 0.177)

Supplementary Materials Table S3. Univariate correlations in all subjects between HDL-mediated macrophage cholesterol efflux (MCE) capacity and aortic diameter, apoA-I, HDLc and BMI.

	**Aortic diameter**	**ApoA-I**	**HDLc**	**BMI**
**Efflux in all subjects**	-0.2 (-0.35 – (-0.043))	0.43 (0.29 – 0.55)	0.39 (0.25 – 0.52)	-0.36 (-0.50 – (-0.22))
**p value**	<0.05	<0.001	<0.001	<0.001
**Efflux in AAA patients**	-0.062 (-0.24 – 0.12)	0.41 (0.24 – 0.55)	0.37 (0.20 – 0.52)	-0.34 (-0.50 – (-0.17))
**p value**	ns	<0.001	<0.001	<0.001

Results expressed as r Pearson coefficient (95 % CI), ns = non-significant

Supplementary Materials Table S4 Multivariate lineal regression of Growth Rate with MCE capacity in small/medium size AAA group adjusted for age, BMI, smoke, statins and DBP

Coefficients[a]			
**Model**	**Standardized coefficients**	**t**	**p value**
	Beta		
Age	-0.118	-1.047	0.299
BMI	-0.197	-1.548	0.126
Smoke	0.172	1.481	0.143
Statins	-0.236	-2.043	0.045
DBP	0.088	0.764	0.448
MEC capacity	0.004	0.027	0.979
Aortic Diameter	0.122	1.029	0.307

a. Dependent variable: Growth rate

biomolecules

MDPI

Supplementary Materials Table S5 Multivariate Cox regression analysis of 5-year predictors of need to surgery in all AAA patients. MCE tertiles were analyzed as a categorical variable using the upper tertile as reference.

All AAA	p value	HR	95% CI for HR	
			Lower	Upper
Upper tertile (ref)	0.043			
Lowest tertile	0.014	3.305	1.268	8.617
Mid tertile	0.029	2.882	1.113	7.467
Current smoking	0.046	0.462	0.217	0.985
CVD	0.137	1.944	0.810	4.668
Use of aspirin	0.270	0.649	0.300	1.400
Use of statin	0.242	0.636	0.298	1.357
Use of ACE inhibitor	0.174	1.701	0.791	3.658
Diastolic BP	0.075	1.026	0.997	1.056
BMI	0.086	0.897	0.792	1.015
Lowest ABI	0.978	0.968	0.098	9.598
Max AAA diameter	0.000	1.106	1.080	1.132

Supplementary Materials Table S6 Multivariate Cox regression analysis of 5-years predictors of need to surgery in small/medium AAA patients. MCE tertiles were analyzed as a categorical variable using the upper tertile as reference.

Small/medium AAA	p value	HR	95% CI for HR	
			Lower	Upper
Upper tertile (ref)	0.622			
Lowest tertile	0.478	1.842	0.341	9.945
Mid tertile	0.330	2.131	0.465	9.775
Current smoking	0.601	0.700	0.184	2.665
CVD	.644	1.545	0.244	9.779
Use of aspirin	0.022	0.152	0.030	0.764
Use of statin	0.141	0.343	0.083	1.425
Use of ACE inhibitor	0.288	2.129	0.529	8.569
Diastolic BP	0.613	1.011	0.969	1.055
BMI	0.801	0.977	0.815	1.171
Lowest ABI	0.793	0.669	0.033	13.562
Max AAA diameter	0.000	1.267	1.129	1.422
